# Introducing the notion of social context of collective trauma to ESTSS

**DOI:** 10.3402/ejpt.v4i0.21258

**Published:** 2013-06-06

**Authors:** Dean Ajduković

**Affiliations:** Department of Psychology, Faculty of Humanities and Social Sciences, University of Zagreb, Zagreb, Croatia

**Keywords:** collective trauma, social context of traumatization

## Abstract

Living amidst war and mass suffering while grasping the opportunity for professional growth, intertwined into my life perspective. Along the years, ESTSS provided a backdrop for my contacts with mental health colleagues from whom I learned, and among whom many became my friends. These rich experiences guided me towards promoting awareness within ESTSS of the importance of social context in which healing of traumatized populations is expected to progress. Each incident of organized violence leaves behind new scores of traumatized individuals and family members, among whom many will need support that may stretch their resources beyond reasonable limits. We need to acknowledge the hindering effects of living in such a social context and that many people that we meet as professionals may carry the burden of unresolved trauma, which should not go by unattended.

Inhabitants of the charming old city of Bergen in Norway told us that June 1993 will be historically remembered because of 6 days of sunny weather in a row. People in the streets were friendly and the fish market displayed dozens of types of smoked salmon at a reasonable price. The fishermen selling their own products generously offered samples, so that one could almost enjoy a free lunch and also learn how the fish was prepared. But this was not why I felt that I was in the right place at the right time. I was a member of a small group of mental health professionals from war-torn Croatia and Bosnia Herzegovina who attended the third European Conference on Traumatic Stress. UNICEF sponsored us because none of us could afford the costs. Colleagues from besieged Sarajevo could not have left the city and return without a UN's “blue pass”. Atle Dyregrov and his colleagues at the Center for Crisis Psychology in Bergen were instrumental in getting us to the conference. On the second day, Atle and his wife Kari hosted a lunch in their home and challenged us to take a swim in the beautiful but freezing cold fjord below their sun deck. Only some of us dared to. At that time, we in Croatia had already been struggling for 2 years trying to provide the best possible help to populations traumatized by war. Our knowledge and skills were very limited, and there were practically no financial resources. The sheer feeling of civil and professional responsibility was the driving force behind the delivery of services under extremely difficult and often dangerous circumstances.

During this conference, at a separate meeting, ESTSS was born, and I am proud to have been a witness to this. Frankly speaking, at that time I was not aware that my future professional life would become closely connected with ESTSS. Over the next 20 years, it proved to be one of my most important networks to meet, learn, work and become friends with a number of outstanding colleagues.

The largest part of my work in the field of trauma started in 1991. In the following years, we dealt with uprooted and traumatized refugees and internally displaced people. Working day after day under threat of bombing, my group of professional colleagues and friends was able to provide psychosocial services to about 3,500 individuals who lived in refugee centers. With devoted colleagues and many committed students of psychology and social work, over the next 5 years we succeeded to deliver a range of interventions that helped stabilize, restore hope and reduce post-traumatic symptoms of these people (Ajduković, 1992, 1993, 1994a, 1994b, 1995, 1996; Ajduković, Čevizović, & Petković, 1996; Ajduković D, & Ajduković, M., 2000, 2003; Ajduković M, & Ajduković, D., 1993, 1998). As we feverishly developed, provided and adapted psychosocial support interventions, I was aware that we also had a responsibility to evaluate the effects of this work (Ajduković, 2008). This was obviously related to my primary training in human experimental psychology and research methods, and my role as university professor of psychology.

When the refugees were able to return to their devastated home communities, we managed to provide outreach support for another 2 years (Ajduković, 1997a; Ajduković M, & Ajduković D., 1996). One of the key lessons for me was the amazing resilience that most of these people demonstrated in the face of ongoing hardship (Francišković et al., 2008). These dramatic experiences shaped my professional career. I was privileged to learn from these traumatized individuals and appreciate how they rebuilt their lives in the devastated communities. However, had it not been for two Norwegian psychiatrists Sigurd Wisloff and Lars Weisaeth, who I met at a conference in Croatia in 1992, I may not have become connected with ESTSS at all a few years later. Sigurd and Lars represented the Norwegian Council for Mental Health, which provided initial financial support to my exhausted group. This was both recognition of the validity of our work and also a boost to continue working. In fact, this prompted us to establish the local non-governmental organization—Society for Psychological Assistance (SPA). Over the next 10 years, SPA became the leading regional non-governmental organization in mental health, trauma and recovery.

Over the course of the past 20 years, I was fortunate to meet and learn from a number of experts in trauma. One of them, Lars Weisaeth, nominated me for the board of ESTSS, which I joined in 1997. My first board meeting was held in Uppsala in conjunction with Psychotraumatology Days organized by Tom Lundin. As I came out from the train station, I saw another person struggling to figure out how to get to the meeting place. This is how I met Ueli Schnyder. In the coming years, our professional paths have, to my delight, crossed many times. Ueli became president of ESTSS and ISTSS. Eventually, I remained on the ESTSS board for 10 years, serving as member, president and organizer of the 10th ECOTS in 2007.

I tried to bring two things to ESTSS’ attention. First, an insight into psychotrauma work in the Balkans and beyond the boarders of European Union. At that time, little was known about professional challenges and efforts of mental health providers in the countries ravaged by the war that followed the breakup of Yugoslavia. Yet, in response to the tremendous needs, mental health professionals and volunteers were struggling to provide care to thousands of highly traumatized people in Croatia, Bosnia Herzegovina, Serbia, Macedonia and Kosovo. Behind the pictures of besieged Sarajevo, shown almost daily on television, there were dozens of heroic psychiatrists, psychologists and social workers who were learning by doing, day-after-day. Training many of them during the mid- and late-1990s is among my most inspiring life experiences. Yes, it was dangerous and uncertain to be flown by NATO military Hercules airplanes in and out of Sarajevo, just to deliver training in basements around the city to colleagues who were eager to learn from our experiences. But it was worth every minute because of such a deep sense of purpose that one experiences so rarely in a lifetime. In return, I was privileged to share these experiences at ESTSS meetings and to lecture in centers of knowledge around Europe (Ajduković, 1994a, 1994b, 1997b, 1999a, 1999b, 2000, 2003, 2004a, 2004b). One of the important concepts that emerged from my work is the “Pyramid of community-based psychosocial services for traumatized populations” (Ajduković, 1997a, 2000; Ajduković D, & Ajduković M., 2003). It is based on the public health approach and captures the relationship between the need for services, allocation of resources and the required level of mental health expertise to provide such services. The same concept was later adapted by leading international expert groups (e.g., Psychosocial Working Group; Inter-Agency Standing Committee [IASC], 2007) but the original authorship seems to have been lost.

Second, I worked to increase awareness of connections between destruction of a community, resulting in collective trauma and social–psychological processes that affect recovery (Ajduković, 2001, 2004c, 2005; Ajduković & Čorkalo, 2004; Ajdukovic D, & Ajdukovic M., 2002; Ajduković D, & Ajduković M., 2003; Bogic et al., 2012; Rooze, De Ruyter, Ajduković, Fundter, & Hövels, 2006). Man-made collective trauma violates human rights. This adds the specific component with long-term consequences regarding the recovery of people and communities that suffered such violations (Priebe, Bogic, Ajdukovic, et al., 2010). Moreover, such collective trauma has distinct qualities that distinguish it from individual traumatization (e.g., traffic accident, rape, assault, life-threatening illness). Typically, there is a political component to it and a need to blame not only the individual perpetrators, but also the group that perpetrators belong to (Biro et al., 2004). Once the notion of collective victimization becomes embedded in the minds of people, blaming for this is attributed not only to individual perpetrators, but it is easily extended towards the whole social group to which they belong to. At the same time, collective guilt is difficult to accept, and individuals tend to feel unjustifiably blamed for things that were done by other people from their group (Jelić, Čorkalo-Biruški & Ajduković, in press). They use denial and other rationalizations to be able to survive with such unpleasant feelings. When people belonging to groups that were in violent conflict continue to live next to each other, remaining tensions have clinical implications for treatment. Illustrative examples are North Ireland, countries of former Yugoslavia, some Baltic States, and tensions in Spain between the former enemies in the civil war 60 years ago. Many years after collective trauma happened, a perceived threat to group identity (whether realistic or symbolic) can serve as a trauma trigger. I argued that psychotraumatology should consider how the social context of traumatization remains relevant for healing (Ajduković, 2004c; Priebe, Bogic, Ashcroft, et al., 2010). Understanding both the past and the current contexts in which recovery should progress is clinically important. My efforts to integrate understanding of consequences of human rights violations and social–psychological processes in which people rebuild their lives after trauma were recognized by colleagues, as I was privileged to give two keynote lectures at ESTSS conferences (Istanbul, 1999; Berlin, 2003).

During the time of my presidency in 2004, we were all shaken by a series of events that highlighted the importance of ESTSS leadership in trauma. These included the Madrid train bombings in March (191 killed), the Beslan school massacre in September (385 killed), and the Asian tsunami in December (almost 9,000 European tourists killed). Once again collective trauma was brought into our homes and work. ESTSS worked with colleagues in Spain, Germany and Scandinavia to help alleviate suffering of the survivors. First experiences were shared at the next European conference in Stockholm (2005). The Scandinavian experiences were most notable for me. They revealed that even in the countries with a long tradition of early trauma interventions, abundant resources and high levels of organizational infrastructure, there were sectors that did not function as expected. These tragic events, again, helped us learn how to better deal with the needs of survivors and the affected communities. They also showed the potential and unique value of ESTSS to support colleagues when their societies are struck by disaster or terrorist attack. However, about the same time we became aware of the disproportionate availability of help and expertise around Europe in such crises. For instance, in the following decade we learned much more about consequences and how they were dealt with in the case of survivors of the tsunami in Scandinavian survivors of the Asian tsunami and terrorist attacks in Madrid and London, than with the aftermath of the school hostage crisis in Beslan in North Ossetia, Russia.

The awareness of uneven distribution of knowledge and access to psychosocial care and post-traumatic stress treatment for victims of disasters and terrorist attacks around Europe, 3 years later resulted in The European Network for Traumatic Stress (TENTS) project (2007–2009). I am happy to have been part of it and took responsibility for five countries (Croatia, Bosnia Herzegovina, Macedonia, Serbia, Slovenia). The project, funded by the European Union, helped to develop a European-wide network of expertise and increased post-disaster evidence-based mental health service capacity. The main tools to achieve this included the mapping of available services around Europe and a systematic review of evidence-based post-traumatic stress interventions. The TENTS guidelines for the delivery of post-disaster psychosocial care in all European countries were produced and a short description of a range of interventions for victims of disaster that are advised according to the guidelines (Bisson et al., 2010; Witteveen et al., 2012). The wide dissemination of these instruments was done as part of the TENTS Training and Practice (TENTS-TP) project between 2010 and 2011 (Pearce et al., 2012). The emphasis was on training trainers to ensure the widest possible implementation of evidence-based practice for those affected by traumatic events that promotes social inclusion throughout the entire European region. The main tools to achieve this were the specifically developed curriculum and educational materials, tailored to different cultures and languages across Europe. I was able to train 93 participants in the five countries that I was responsible for. In turn, they trained hundreds of care providers in the following years. Miranda Olff and Jonathan Bisson directed both these projects, which for me was a valuable learning opportunity about leadership in multinational projects.

Being part of ESTSS leadership was sometimes demanding but always very gratifying. During my presidency (2003–2005), several processes started that led to the organization's transformation. During the previous years, there was ongoing discussion about fair regional representation on the board of directors due to rapidly emerging interest for traumatic stress in countries beyond the traditional centers, such as United Kingdom, The Netherlands and Scandinavia. Another issue was securing financial stability of the organization. Our growing concern was how to involve more ESTSS members in governance of the society. As a consequence, we started the democratization of the election process. Kerstin Bergh Johannesson and Oscar Daly led this effort, which eventually led to establishing the practice of full involvement of the membership in voting for the board members. The work on defining the training standards in psychotrauma was chaired by Karen Sadlier. After several years of impasse, ESTSS was able to develop standards of training eventually leading to the certificate in psychotraumatology. We were also concerned about how to maintain ESTSS’ leadership in promoting psychotrauma work throughout Europe, and at the same time support the emerging national and regional initiatives. This resulted in guidelines for affiliating other societies with ESTSS (led by Ufuk Sezgin and I) and ensuring regional representation on the board. This was an attempt to balance the fact that some European regions were the leaders in trauma work, with the fact that other regions also needed a voice in the ESTSS leadership. However, the most important impetus towards restructuring ESTSS came from Berthold Gersons. Berthold took over presidency in 2005 from me, and successfully navigated the society into becoming the umbrella organization as we know it today. He remained my model and inspiration not only in how to “think out of the box”, but also in the unobtrusive, yet determined way to achieve high goals and make other people embrace his ideas. For me, this is a fine example of transformative leadership. I am also proud that I persuaded Brigitte Lueger-Schuster to run for the ESTSS board. We first met at a conference on psychosocial acute care in emergency situations, which she organized in Vienna (2000). We became friends who share many values and interests on the crossroad of trauma and human rights. I am very impressed with her studies of long-term consequences of trauma experienced by Austrian children during World War II and abuse of children by Catholic priests.

I like organizing conferences (and people say that I am good at it!). It is always a challenge. On one side, there are (manageable) risks related to attractiveness of the program, finances and organizational issues. On the other side, this is a legitimate way to bring friends together for a few days to meet, discuss, plan and enjoy. Apart from several other world and European conferences, I am proud to have organized two ESTSS events. One was the largest ESTSS regional meeting (Zagreb, 2004). It attracted over 230 participants from 10 countries, with an emphasis on clinical training and workshop format. The second was the 10th European Conference (Opatija, 2007). There we introduced a new format that was very well received: Key Controversies Debate during which two prominent psychotraumatologists confront views on a core issue in our discipline. This format proved attractive in the following ESTSS biannual conferences. A lot of people still tell me that they remember the Opatija conference for its wonderful atmosphere, hospitality and food. After all, experience shows that apart from a few groundbreaking presentations, what people take home from conferences are the overall impressions and new contacts. The conference in Opatija still remains the only ESTSS conference held outside the European Union, which will hopefully change in the near future. I am also honored to have served as program chair or scientific committee member for the 11th and 12th conferences (Bologna, 2013; Vienna, 2011).

I already recognized that my work in the field of psychological trauma evolved and expanded over the past 20 years due to my close association with ESTSS. Now I would like to point to several new areas that we should look into in the near future.

In the past several years, I became increasingly concerned with prevention of violence, not only in helping the people who are traumatized by violence. I got involved in the treatment of perpetrators of family violence, peer violence in children and adolescents and prevention of violence in adolescent romantic relations. Along the way, I became more aware how often perpetrators have a history of their own trauma. My experience shows that many perpetrators of family violence have been traumatized themselves, typically during child and youth formative years. Yet, this goes unrecognized since the penal and rehabilitation systems focus primarily on the consequences of breaking the law. However, the needs of perpetrators of violence are twofold: they need to develop self-control and self-awareness competencies. At the same time, they also need acknowledgement of their own suffering and to receive treatment as victims of trauma.

Another one of my concerns is that so many people live with disturbing consequences of trauma that go unrecognized and therefore unaddressed. For example, after each attack and retaliation in the Middle East, I think about the dozens of new people who have just suffered trauma. I also refer to thousands of people whose traumatic experiences did not have the legitimacy to surface during their life-time because they felt that they had no right to receive help for consequences of own traumatic distress. These include people who were on the “wrong side” in the conflicts and wars because their country or the group subscribed to the military or racist ideology, and many individuals committed crimes on behalf of the ideology or the ethnic group. Yet, among these people there are many, many ordinary, civilian individuals who live burdened by the stigma of belonging to the aggressor group. Hence, they were not seen as having a “legitimate right to suffer from trauma” and consequently did not have access to help. Their children and grandchildren may live in the family atmosphere of unrecognized victimhood, which is co-conductive to the trans-generational transmission of trauma.

In the coming years, I would like to see ESTSS lead the way in raising awareness of the presence of concealed and delayed effects of earlier trauma on the people who are, for whatever reason, reluctant to seek help. It will be important for ESTSS to continue to advocate for and disseminate evidence-based knowledge and practices in parts of Europe that lack all types of resources for quality trauma work. In my view, it will be essential for ESTSS to spearhead the effort to train more care providers in different professions for the benefit of trauma survivors presenting at different services and entrance points into the systems. Finally, I hope to see increased awareness of relations between interpersonal and intergroup violence, violation of human rights and suffering trauma on one hand, and more attention devoted to long-term consequences of collective trauma for a community and affected society.
